# Does teaching non-technical skills to medical students improve those skills and simulated patient outcome?

**DOI:** 10.5116/ijme.58c1.9f0d

**Published:** 2017-03-29

**Authors:** Vera Hagemann, Frank Herbstreit, Clemens Kehren, Jilson Chittamadathil, Sandra Wolfertz, Daniel Dirkmann, Annette Kluge, Jürgen Peters

**Affiliations:** 1 Business and Organizational Psychology, Faculty of Psychology, Ruhr-University Bochum, Germany; 2 Clinic for Anesthesiology and Intensive Care, University of Duisburg-Essen and University Hospital Essen, Germany; 3 Business Psychology, Faculty of Engineering, University of Duisburg-Essen, Germany

**Keywords:** Non-technical skills, reliable teamwork, medical students, human patient simulation, teamwork-relevant attitudes

## Abstract

**Objectives:**

The purpose of this study is to evaluate the effects of a tailor-made, non-technical skills seminar on medical student’s behaviour, attitudes, and performance during simulated patient treatment.

**Methods:**

Seventy-seven students were randomized to either a non-technical skills seminar (NTS group, n=43) or a medical seminar (control group, n=34). The human patient simulation was used as an evaluation tool. Before the seminars, all students performed the same simulated emergency scenario to provide baseline measurements. After the seminars, all students were exposed to a second scenario, and behavioural markers for evaluating their non-technical skills were rated. Furthermore, teamwork-relevant attitudes were measured before and after the scenarios, and perceived stress was measured following each simulation. All simulations were also evaluated for various medical endpoints.

**Results:**

Non-technical skills concerning situation awareness (p<.01, r=0.5) and teamwork (p<.01, r=0.45) improved from simulation I to II in the NTS group. Decision making improved in both groups (NTS: p<.01, r=0.39; control: p<.01, r=0.46). The attitude ‘handling errors’ improved significantly in the NTS group (p<.05, r=0.34). Perceived stress decreased from simulation I to II in both groups. Medical endpoints and patients´ outcome did not differ significantly between the groups in simulation II.

**Conclusions:**

This study highlights the effectiveness of a single brief seminar on non-technical skills to improve student’s non-technical skills. In a next step, to improve student’s handling of emergencies and patient outcomes, non-technical skills seminars should be accompanied by exercises and more broadly embedded in the medical school curriculum.

## Introduction

According to an evidence-based estimate, 400,000 preventable patient deaths occur annually in US hospitals.^1^ In Germany, preventable deaths in hospitals are five times more frequent than deadly traffic accidents, and up to 80% of adverse events in anaesthesiology are attributed to human errors, including team performance breakdown.^2^ For reliable team performance and to minimise human errors in medicine and other domains such as aviation, non-technical skills (NTS) are essential.^3^^-^^5^ Feedback based on NTS rating systems for evaluating trainee surgeons' NTS and for improving performance, for example, are perceived as highly valuable and useful by trainees and supervisors.6 Accordingly, interventions such as “aeromedical” and “anaesthesia crisis resource management” have been developed to improve teamwork-relevant NTS (e.g., communication, decision making, situation awareness, or adaptation) and to increase team performance and safety in high-responsibility teams (HRTs).^5^^,^^7^^-^^9^ NTS comprise an individual’s cognitive, attitudinal, and social skills that supplement the individual’s task work-related expertise.^5^ NTS-oriented interventions are instructional strategies for HRTs to a) train the usage of all available resources efficiently (i.e., humans, equipment, and information), b) enhance teamwork and thereby performance, and c) diminish the likelihood of possible human error to mitigate consequences for humans or the environment.^10^

Study findings have supported the effectiveness of NTS-oriented interventions in augmenting teamwork competencies, e.g., in aviation, military, fire service, or medical teams on their reactions, teamwork safety-relevant attitudes, knowledge, and behaviour.^5^^,^^10^^-^^12^ For example, a meta-analysis by Salas and colleagues reported positive effects of NTS interventions on team member’s reactions and teamwork safety-relevant attitudes.^13^  A meta-analysis conducted by O`Connor and colleagues found support for these effects.^12^ The reported studies demonstrated positive effects of NTS interventions on team member’s reactions, teamwork safety-relevant attitudes and behaviour. Medium-sized effects were found concerning safety-relevant knowledge gain.

Positive effects of teamwork competencies on team performance have also been demonstrated, with medium to large effect sizes found regarding the positive effects of team process behaviours on clinical performance measures such as task management, surgical complications, operating time, or patient morbidity.^14^ Thus, NTS interventions support teamwork-relevant competence acquisition, and teamwork competencies positively influence clinical performance.

In a novel attempt to unify their curricular targets, German medical faculties have recently implemented the teaching of non-technical skills, and the German Association for Medical Education has recently published a “Learning Objective Catalogue for Patient Safety”.^15^ However, limited data exists regarding the suitability and effectiveness of NTS interventions for medical students.

To assess positive seminar effects on teamwork competencies for this target group, the widely used training evaluation hierarchy from Kirkpatrick (1998) is applied.^16^ This hierarchy categorises training outcomes on four levels. The first level comprises the evaluation of “reactions”, such as subjectively perceived enjoyment and perceived usefulness of the NTS seminar. The second level is “learning”, and encompasses the participant’s attitudinal changes and knowledge gain after an NTS seminar. The third level is “behavioural changes”, and refers to the application of acquired knowledge and skills to the job. The fourth level is “results”, and refers to the benefits for the organisation, such as successful patient treatment.

In light of the above, we investigated the effects of a single short NTS-oriented seminar on medical student’s reactions, teamwork, safety-relevant attitudes, and non-technical skills as well as on simulated patient’s outcome. Based on studies from various occupational domains that found positive changes in team member’s NTS after receiving NTS training, we assume that medical students will also show a positive change concerning their NTS after receiving an NTS seminar, whereas a medical seminar control group will not show this improvement.^5^^,^^8^^,^^10^^-^^13^

Hypothesis 1: Medical students who receive an NTS seminar will have higher values concerning their NTS after the seminar than before the seminar.

Based on previous study findings that demonstrated positive changes in team members’ safety-relevant attitudes after receiving NTS training, we also assume that medical students will show a positive change concerning their teamwork safety-related attitudes after receiving an NTS seminar, whereas the medical seminar control group will not show this improvement.^9^^-^^13^

Hypothesis 2: Medical students who receive an NTS seminar will have higher values concerning their teamwork safety-relevant attitudes after the seminar than before the seminar.

As positive effects of teamwork competencies and NTS on team performance (e.g. surgical complications, patient morbidity) have been demonstrated, we also assume that teaching NTS will lead to a more successful patient treatment after the NTS seminar, as evidenced by a comparison between the NTS seminar group and the medical seminar control group.^14^

Hypothesis 3: Medical students who receive an NTS seminar will show a better performance in managing a simulated patient than those who receive a medical seminar.

## Methods

This study explored the effects of an NTS seminar on student’s behaviours during simulations as well as medical endpoints and simulated patient’s outcome. Additionally, we analysed changes in teamwork-supporting attitudes and perceived stress during both simulation scenarios. Data was collected from questionnaires, observations and the simulator software.

### Study design and participants

This was a randomised, double-blind study with a pre-test-post-test design investigating the usefulness of NTS seminars within medical education. It was randomised because at the beginning of the semester, students were randomly assigned to either an NTS seminar group or a medical seminar group by Dean’s department. It was double-blind as the students were unaware of their group allocation. Moreover, the investigators were also unaware of the student’s group allocation when they conducted behavioural assessments during the simulations. The design was pre-test-post-test as we used a baseline measurement at the beginning of the study and a follow-up measurement after the NTS seminar. Thus, we were able to analyse effects over time. Based on the comparison between the NTS seminar group and the medical seminar group, we were also able to analyse between-group effects.

One hundred and four 4th-year medical students participating in a two-week course on emergencies taught by the Department of Anaesthesiology & Intensive Care Medicine were initially enrolled and randomly allocated to either an NTS seminar group or a control group receiving a traditional medical seminar. Participation in the study was voluntary and had no influence on successful participation in the two-week course. We obtained oral and written informed consent from each participant, and all students received the complete course contents regardless of study participation.

Thirteen participants had to be excluded from the analysis as they did not attend one of the simulations, and a further fourteen participants were excluded as they either did not attend one of the seminars or did not complete all questionnaires. Therefore, data from seventy-seven participants were ultimately analysed. The students’ mean age was 25.9 years (±3.5 SD, range: 21 to 39) and thirteen students had previous experience working in the field of medicine (eight in nursing and five in emergency services).

**Figure 1:**
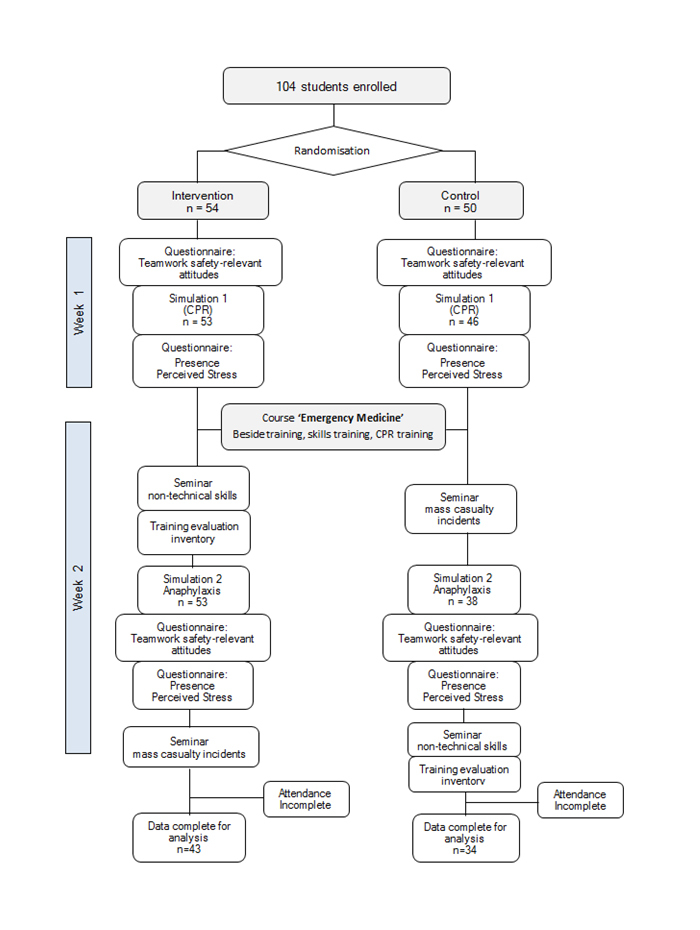
Outline and timeline of experimental set-up

All participating students completed the same simulated resuscitation scenario at the beginning of the study, which served as baseline measurement in week one. The following week, they attended either a 90-minute seminar entitled “Factors influencing successful teamwork” (NTS group) or a medical seminar unrelated to the non-technical skills to be tested (“concepts for mass-casualty incidents”, the control group, CG). On the subsequent two days, students were tested in a second simulation (post-test measurements), an anaphylactic shock scenario. To ensure that no student missed any lectures and no knowledge gaps existed, after study data sampling, students of the control cohort attended the NTS seminar, while the NTS group was instructed in mass-casualty incidents. The experimental design is depicted in Figure 1.

In detail, students were welcomed individually in week one and asked to complete a questionnaire measuring teamwork safety-relevant attitudes (simulation scenario I at baseline). Afterwards, each student was accompanied to the simulation room and familiarised with the team (medical assistants performing to predefined standards) and equipment and received standardised scenario information. Each student was observed and videotaped through a one-way transparent window from a second room in order to assess NTS and key medical interventions at baseline. After the simulation scenario, students completed a second questionnaire measuring presence and perceived stress in simulation I. Before leaving, students received a short debriefing regarding their performance. In week two, NTS-group participants attended the NTS seminar (CG attended the medical seminar). At the end of the seminar, each participant filled in the Training Evaluation Inventory for assessing the seminar. On the following days, students participated in the second simulation scenario (post-test measurement) and were observed and videotaped again (same procedure as in simulation I). Subsequently, students completed questionnaires measuring the presence and perceived stress in simulation II and the teamwork safety-relevant attitudes. Again, students received a short debriefing.

The study was conducted at the Department of Anaesthesiology & Intensive Care Medicine, University Hospital Essen, University of Duisburg-Essen, Germany and was approved by the University’s local institutional ethics committee in Essen in September 2014.

### Treatment: the NTS seminar design

In week two, students attended a 90-minute NTS seminar entitled “Factors influencing successful teamwork”, led by the same two instructors (two authors) throughout the course. The seminar aimed to sensitise students for teamwork-supporting behaviour and attitudes towards leadership and assertiveness as well as dealing with mistakes and stress.^17^ Topics covered included situation awareness^18^ and its impact on teamwork, as well as shared mental models and strategies to improve them (e.g., loud verbalisation during work or debriefings) as prerequisites for team coordination,^19^ communication as a prerequisite for coordination (e.g., clear pronunciation and reconfirmation),^20^ and feedback rules. Students were also familiarised with possible obstacles which might impair communication such as perception and selectivity, and discussed rules for successful communication. The final exercise was a type of demonstration-based learning technique considering observational learning processes.^21^^,^^22^ “Observational (or demonstration-based) learning is the process of acquiring knowledge, skills, and attitudes (KSAs) through viewing examples of performance”.^21^ The exercise required the students to point out successful and less successful behaviours regarding reliable teamwork based on a video demonstrating teamwork in a patient-handover situation between hospital staff and a helicopter emergency medical service. The critical cues from the video were subsequently discussed and the previously acquired competencies concerning the teamwork-supporting behaviour and attitudes had to be consolidated. Successful and less successful behaviours were chosen in the demonstration, as positive and negative models support the generalization of the targeted behaviours.^23^

### Data collection methods

#### The Simulations

A human patient simulator was used for assessing the student’s behaviour. It was placed in a mock patient wardroom next to a bed-ridden “roommate” (manikin). With its integrated physiology software, this simulator allows for realistic scenarios adjusted to medical actions such as endotracheal intubation, defibrillation, or drug administration. Patient’s comments, sounds, and vital signs were loaded wirelessly while running the scenario. All medication and equipment required for managing the simulated patient were provided. The scenarios were recorded using an audio-visual recording system, which captures physiological data including event logs, waveform displays, and annotations. Scenarios were developed and programmed by three of the authors with a strong background in handling emergencies. One author operated the simulator, the recording system and technical facilities.

In simulation I (10 minutes duration, pre-test measurement), a cardiovascular risk patient with sudden unconsciousness and ventricular fibrillation was portrayed and cardiopulmonary resuscitation (CPR) according to ERC guidelines was required. The simulator was programmed for a return of spontaneous circulation after the 4th defibrillation attempt if correct CPR had been performed and adrenaline and amiodarone had been administered.

In simulation II (10 minutes duration, post-test measurement) a young patient recovering from surgery for radial fracture was portrayed.  Here, an anaphylactic reaction to an antibiotic infusion was presented, which progressed to anaphylactic shock if not treated early with adrenaline. Specifically, the simulation was escalated every 3 minutes to increasing symptoms (stage 1: coughing, itching; stage 2: worsening to tachycardia and hypotension; stage 3: overt shock). Treatment with adrenaline was necessary to improve the simulated patient’s condition.

#### Non-Technical Skills

The Anaesthesia Non-Technical Skills (ANTS) observation system, including the four categories situation awareness, task management, team working, and decision making, was applied to assess the students’ NTS during simulations I and II.^24^^,^^25^ The modified instrument for assessing Danish anaesthesiologist’s NTS (ANTSdk) was not applied, as the authors stress the cultural specificity of this instrument. Denmark is considered a feminine society by Hofstede, whereas Germany is not.^26^ Each category consists of 3-5 elements characterised by various behavioural markers for good and poor performance (see Appendix A). The behavioural markers were adapted to the simulation scenarios.

Two observers rated every student’s behaviour. Due to the substantial number of behavioural markers, each observer rated half of them. All elements and categories were rated on a four-point scale from 0 to 3, i.e., from poor (performance endangered or potentially endangered patient safety, serious remediation required) to good (performance of consistently high standard, enhancing patient safety; could be used as a positive example for others).^25^ Element ratings were averaged, resulting in ratings for each category.

#### Medical Skills

The completion of key treatments and their timing was recorded for each scenario (see Appendix B), e.g. “call for assistance”, “application of oxygen”, “time to first chest compression”, “time to adrenaline administration”, and “time to defibrillation”. The relevant outcomes were “return of spontaneous circulation” (yes/no) and “resolution of anaphylaxis (yes/no)” in simulations I (CPR) and II, respectively.

#### Teamwork Safety-Relevant Attitudes

A 20-item questionnaire was applied before simulation I and after simulation II with a five-point Likert scale ranging from 0 (total disagreement) to 4 (total agreement). The questionnaire was tailored to the target cohort and based on established instruments.^9^^,^^27^^-^^29^ It covers eight of the most frequently investigated safety-relevant attitudes, i.e. command roles and responsibilities (four items, e.g., ‘Team members should not question the decisions or actions of senior staff’, α=.72), speaking up (two items, e.g., ‘I inform other team members when my workload is too high’, α=.56), debriefing (two items, e.g., ‘A regular debriefing of procedures and decisions after an emergency care is an important part of teamwork’, α=.42), feedback and critique (two items, e.g., ‘Disagreements in the team are appropriately resolved, i.e., it is not ‘who’ is right but what is best for the patient’, α=.21), realistic appraisal of stress (three items, e.g., ‘Personal problems can adversely affect my performance’, α=.76), denial of stress (three items, e.g., ‘A professional doctor can hide personal problems during the whole emergency care’, α=.71), handling errors (two items, e.g., ‘I am more likely to make errors in tense or hostile situations’, α=.69), and teamwork (two items, e.g., ‘I enjoy working in a team’, α=.40).

#### Presence within the Simulation Scenario

The instrument “Presence for lab-based microworld research” (PLBMR) was used to measure the immersion in the two scenarios.^30^ The questionnaire uses a six-point Likert scale from 0 (is not true) to 5 (is fully true). An example item is ‘I felt like I was part of the simulation context’ (Cronbach’s α=.83).

#### Perceived Stress

Five items were developed to measure perceived stress (five-point Likert scale from 0 (total disagreement) to 4 (total agreement), e.g. ‘In retrospect, during the simulation scenario I worked up a sweat’, Cronbach’s α=.85).

#### Training Evaluation Inventory

The 17-item Training Evaluation Inventory (TEI) was applied to assess the student’s reactions and subjectively rated learning success after the NTS seminar (five-point Likert scale from 1 to 5).^31^ It covers seminar outcomes based on the reaction level, which included reported enjoyment (three items, e.g., ‘I enjoyed learning’, α=.8), perceived difficulty (three items, e.g., ‘I understood all technical terms’, α=.69), and perceived usefulness (four items, e.g., ‘The training is useful for my profession’, α=.93).10,32-33 Additionally, the learning level of Kirkpatrick’s levels of evaluation is assessed.^16^ Learning is divided into knowledge (three items, subjectively rated learning success, e.g., ‘I think my knowledge has been expanded in the long term’, α=.66) and attitudes (three items, e.g., ‘I would recommend this training to my colleagues’, α=.87).

### Data analysis

To ensure that the NTS group and control group did not differ before the seminar intervention, t-tests for unpaired samples were performed to detect significant differences in demographic or study variables. The prerequisites concerning normal distribution and homogeneity of variance (Levene’s test) for the scales and groups were met. To analyse changes over time and between groups regarding NTS and attitudes, univariate analyses of variance with repeated measures were conducted. To analyse differences between groups concerning simulated patient outcome, continuous values were analysed by t-tests. If the assumption of normality was not met, the Mann-Whitney U test was conducted. Categorical values (yes/no) were compared using a Chi-squared test. Analyses were performed using SPSS, Version 23.0, and statistical significance was assumed using an a priori alpha error less than 0.05. 

## Results

Before presenting the results of the hypothesis-testing, descriptive data of study variables are reported as well as the level of significance, to demonstrate that the study results are not systematically influenced by previous differences between groups. The NTS seminar group and the control group were comparable, as no significant differences in age, semester, presence, stress, attitudes, and NTS at baseline (simulation I) were revealed, as depicted in Appendix C.

### Treatment checks

Presence and perceived stress during simulations: Group means and standard deviations during simulation I (pre) and II (post) as well as the average over both groups (total) are displayed in Table 1. Both groups showed a moderate presence during both simulations (pre and post), without significant differences between groups (p=.11), and the presence remained stable over time, as no changes from pre to post were detected (p=.94). Both groups also showed a moderate level of stress during simulations I and II, without significant differences between groups (p=.10). However, perceived stress changed over time (F_(__1,75)_ = 10.11, p<.01, η^2^p=0.12): T-tests for dependent samples revealed a significant decrease from pre to post for the NTS group (t_(42)_ = 2.19, p<.05, r=0.32) and the control group (t_(33)_ = 2.26, p<.05, r=0.37), with a small to medium effect size.

Subjectively perceived outcomes of the NTS seminar were assessed by applying the Training Evaluation Inventory scales regarding reaction and learning. The students assessed their enjoyment (M=3.90, SD=0.85), their perceived usefulness of the seminar (M=3.98, SD=0.94) and their acquired knowledge (M=3.75, SD=0.67) rather positively and considered the seminar to be easy to follow (M=1.37, SD=0.42). Only the attitudes towards the contents of the seminar (M=2.58, SD=0.71) were evaluated as moderate to negative. Overall, the seminar was positively evaluated.

**Table 1:** Mean values and standard deviations regarding presence and perceived stress concerning simulation I (pre) and II (post)

Groups		Presence			Stress	
		M	SD		M	SD
Total	pre	3.41	±1.02		2.82	±0.49
	post	3.40	±1.06		2.58	±0.56
NTS group	pre	3.55	±1.02		2.75	±0.55
	post	3.54	±1.07		2.51	±0.62
Control group	pre	3.23	±1.00		2.90	±0.39
	post	3.22	±1.05		2.68	±0.46

Note: Scale ranges from 0 to 5 for the presence and from 0 to 4 for stress.

### Testing Hypothesis 1: Impact of NTS seminar intervention on non-technical skills during simulations

The results address whether the NTS of the NTS seminar group improved from simulation I (before seminar intervention, pre) to simulation II (post), compared to the control group. Mean values and standard deviations for all four NTS categories for both groups and both performance assessments (pre and post) are displayed in Table 2.

Regarding situation awareness, the main effect for time of measurement was significant, with situation awareness improving significantly from simulation I to II (F_(1,75)_ = 12.83, p<.01, η^2^p=0.51). Situation awareness improved only in the NTS group (t_(__42)_ = -3.74, p<.01, r=0.50). Team working improved significantly from pre- to post-test (F_(__1,75)_ = 11.19, p<.01, η^2^p=0.13). The parameter estimation for simulation II (post) showed a significant difference between the NTS and control group (p<.05). T-tests for dependent samples showed a significant improvement from pre to post only for the NTS group (t_(__42)_ = -3.27, p<.01, r=0.45). Regarding task management, no significant effects were found. Regarding decision making, the main effect for time of measurement was significant, with decision making improving significantly from pre to post (F_(1,75)_ = 16.48, p<.01, η^2^p=0.18). Results of t-tests for dependent samples showed that both the NTS group (t_(__42)_ = -2.75, p<.01, r=0.39) and the control group (t_(33)_ = -3.00, p<.01, r=0.46) improved significantly. Thus, hypothesis 1 was predominantly supported, as three NTS changed significantly from simulation I to simulation II within the NTS group.

### Testing Hypothesis 2: Impact of NTS seminar intervention on teamwork safety-relevant attitudes

Data for ‘command roles and responsibilities’, ‘speaking up’, ‘debriefing’, ‘feedback and critique’, ‘realistic appraisal of stress’, and ‘denial of stress’ showed no significant effects between or within groups over time (.06<p<.81) (see Table 2). The attitude concerning ‘Handling errors’ improved significantly within the NTS group from simulation I to II (t_(__42)_ = -2.33, p< .05, r = 0.34), and did not improve overtime in the control group (t_(33)_ = -0.90, p=.37, r=0.15). ‘Teamwork’ decreased significantly over time in the NTS group (t_(__42)_ = 2.26, p = .03, r = 0.33), but not in the control group (t_(33)_ = 1.15, p=.26, r=0.20). Means and standard deviations for all attitudes for both groups and both simulations (pre and post) are provided in Table 2. Thus, hypothesis 2 was not supported, as only one significant positive attitude change was detected within the NTS group.

**Table 2:** Non-technical skills (NTS) and attitudes for simulation I (pre) and II (post)

Groups	Pre/ post	NTS		Attitudes	
		Means	SD	Means	SD
		Situation awareness		Command roles and responsibilities	
NTS group	pre	1.17	±0.50	3.23	±0.50
	post	1.47	±0.48	3.24	±0.43
Control group	pre	1.18	±0.43	3.23	±0.34
	post	1.33	±0.58	3.38	±0.44
		Team working		Speaking up	
NTS group	pre	1.37	±0.57	2.81	±0.71
	post	1.63	±0.36	2.85	±0.60
Control group	pre	1.31	±0.53	2.75	±0.68
	post	1.43	±0.43	2.82	±0.57
		Task management		Debriefing	
NTS group	pre	1.40	±0.65	3.71	±0.40
	post	1.50	±0.52	3.57	±0.52
Control group	pre	1.30	±0.56	3.56	±0.53
	post	1.43	±0.54	3.59	±0.54
		Decision making		Feedback and critique	
NTS group	pre	0.90	±0.58	3.63	±0.54
	post	1.15	±0.48	3.58	±0.76
Control group	pre	0.85	±0.58	3.59	±0.56
	post	1.15	±0.49	3.76	±0.50
				Realistic appraisal of stress	
NTS group	pre	-	-	2.76	±0.86
	post	-	-	2.88	±0.82
Control group	pre	-	-	2.74	±0.59
	post	-	-	2.54	±0.84
				Denial of stress^*^	
NTS group	pre	-	-	1.92	±0.99
	post	-	-	1.78	±0.95
Control group	pre	-	-	1.81	±0.78
	post	-	-	1.79	±0.91
				Handling errors	
NTS group	pre	-	-	2.49	±0.87
	post	-	-	2.70	±0.69
Control group	pre	-	-	2.76	±0.63
	post	-	-	2.87	±0.56
				Teamwork	
NTS group	pre	-	-	2.70	±0.63
	post	-	-	2.51	±0.69
Control group	pre	-	-	2.71	±0.64
	post	-	-	2.60	±0.69

Note: Scale for assessing NTS ranges from 0 to 3; and scale for assessing attitudes ranges from 0 to 4; *Low values indicate a positive attitude.

### Testing Hypothesis 3: Impact of NTS seminar intervention on simulated patient outcome

Student`s management skills during a resuscitation scenario I (before the seminars) and an anaphylactic shock scenario II (after the seminars) were assessed using videotapes of 154 simulations (77 students experiencing both scenarios) using predefined variables and endpoints, e.g., “time until calling a chief resident or a resuscitation team” or “time until return of spontaneous circulation”.  All results (either mean values and standard deviations or percentage, as well as levels of significance) are presented in Table 3. The groups were comparable at baseline (scenario I), as no significant differences between the groups were detected. A comparison of results for simulation II did not reveal a significant intervention effect on simulated medical outcomes. Hypothesis 3 was therefore not supported. Neither the variable “patient´s condition improved” nor “time until patient´s condition was improved” differed between groups following the intervention (Table 3). In the anaphylactic shock scenario, 60.5% (26 of 43) and 61.8% (21 of 34) of simulated patients died in the NTS and control group, respectively, indicating that the scenario had been properly calibrated to detect intervention effects if present.

## Discussion

This study demonstrates a positive impact of a single NTS seminar on student’s NTS since only the student group that had received the NTS seminar improved significantly from simulation I to II. Nevertheless, this did not translate into the students’ teamwork safety-relevant attitudes, and no statistically significant benefit was apparent regarding simulated patient’s medical outcome.

Overall, the students perceived the simulations as quite realistic and not too stressful. The NTS seminar was well accepted, with the students rating their enjoyment and the perceived usefulness very positively. Although they did not express a great deal of liking for the seminar contents, this is not especially important regarding competence acquisition, as the perceived usefulness is the most important predictor of motivation and intention to apply learned knowledge and skills at work.^31^^,^^32^ The students extended their knowledge regarding safety-relevant teamwork competencies, as 75% of the NTS items improved significantly in students who had received the 90-minute NTS seminar, including a demonstration-based learning approach. On decision making, the control group’s behaviour also improved significantly from simulation I to II. Since students are not yet experts and still have to acquire technical knowledge, the effects of the NTS seminar on their NTS are even more remarkable. In contrast, no significant influence of the NTS seminar on teamwork safety-relevant attitudes was found between groups or over time. However, the fact that not all assessed items showed significant results is not unusual, and many studies addressing the impact of an NTS seminar on attitude changes have failed to find significant effects.^34^

Our results show that a change in the student’s behaviour, the NTS, occurred without concurrent changes in their attitudes. Thus, Kirkpatrick’s assumption of the hierarchy of the four levels of evaluation is again disproved.^35^

In this study, we also tried to assess whether the NTS seminar was able to affect simulated patient’s medical outcomes due to better coordination and leadership behaviour during teamwork, the fourth level in Kirkpatrick’s model of evaluation. While simulated patient outcomes (e.g. variables such as “patient´s condition improved” or “time until patient´s condition was improved”) did not differ between groups in simulation II and were, therefore, unaffected by the NTS seminar intervention, we observed deficits regarding medical treatment in both groups. About 60% of simulated patients died in both groups. This lack of effect might be due to the students` limited previous practical training to manage emergencies. Alternatively, the medical tasks required to treat simulated patients in our scenarios successfully might have been too complex for the students, although 4th-year medical students should have already acquired the proper theoretical knowledge. Since the theoretical knowledge did not seem to have been transferred to practice, the students’ limited abilities may have masked any effect of the acquired NTS on simulated patient treatment.

**Table 3:** Results of comparison between NTS group and control group regarding performance of treatment steps in both simulation scenarios

Resuscitation scenario I			
Treatment steps	NTS Group	Control Group	Significance
Call for help (yes/no)	Yes: 51.20% No: 48.80%	Yes: 35.30% No: 64.70%	χ^2^(1) = 1.94, p = .16
Return of spontaneous circulation (yes/no)	Yes: 39.50% No: 60.50%	Yes: 32.40% No: 67.60%	χ^2^(1) = 0.42, p = .52
Time until emergency call(s)	303.00 ± 39.90	335.80 ± 41.20	U = 120.00, z = -0.43, p = .68
Time until first defibrillation(s)	184.80 ± 8.60	178.40 ± 11.20	U = 698.50, z = .72, p = .47
Time until return of spontaneous circulation(s)	435.00 ± 26.80	466.00 ± 21.50	t_(26)_ = 0.83, p = .41
Anaphylactic shock scenario II			
Treatment steps	NTS Group	Control Group	Significance
Call for help (yes/no)	Yes: 46.50% No: 53.50%	Yes: 23.50% No: 76.50 %	χ^2^ (1) = 4.33, p = .04
Provided oxygen therapy via non-rebreathing mask (yes/no)	Yes: 93.00% No: 7.00%	Yes: 94.10% No: 5.90%	χ^2^ (1) = 0.04, p = .85
Adrenaline administration (yes/no)	Yes: 48.80% No: 51.20%	Yes: 64.70% No: 35.30%	χ^2^ (1) = 1.94, p = .16
Antihistamine administration (yes/no)	Yes: 69.80% No: 30.20%	Yes: 67.60% No: 32.40%	χ^2^ (1) = 0.04, p = .84
Initiation of blood volume expansion therapy (yes/no)	Yes: 55.80% No: 44.10%	Yes: 67.60% No: 32.40%	χ^2^ (1) = 1.12, p = .29
Patient´s condition improved (yes/no)	Yes: 39.50% No: 60.50%	Yes: 38.20% No: 61.80%	χ^2^ (1) = 0.01, p = .91
Time until emergency call(s)	318.40 ± 34.40	356.90 ± 49.90	t_(27)_ = 0.60, p = .55
Time until oxygen administration(s)	152.30 ± 17.20	153.50 ± 18.90	U = 623.50, z = 0.41, p = .69
Time until adrenaline administration(s)	322.50 ± 20.80	336.60 ± 27.30	t_(41)_= 0.41, p = .69
Time until antihistamine administration(s)	247.20 ± 18.70	189.60 ± 13.90	U = 470.50, z = 2.26, p = .02
Time until initiation of blood volume expansion therapy(s)	219.00 ± 21.00	285.00 ± 21.00	U = 167.50, z = -2.31, p = .02
Time until patient´s condition improved(s)	273.10 ± 13.70	241.80 ± 14.30	t_(28)_= -1.56, p = .13

Although no significant impact on patient outcomes was observed under the experimental conditions chosen, our results are nevertheless meaningful, as such analyses are often missing in non-technical skills training research.^5^ Possibly, if simulation training were extended and broadly integrated into the student’s medical curricula, NTS seminars might be more effective.

As the NTS seminar, with duration of 90 minutes, was a rather short intervention, large effects might not be expected. Furthermore, it was a knowledge-based NTS seminar with demonstrated examples, and its learning goals were to enhance the student’s knowledge about teamwork-relevant skills. From training research, it is well known that knowledge-based interventions do not, or only minimally, influence behaviour compared to demonstration-based or practice-based interventions.^36^ It is therefore recommended that future studies analyse NTS seminars of longer duration (possibly over two or more days) combined with practice sessions to achieve both behavioural learning goals and a transfer concerning patient treatment. Such simulations should be used not only to assess the student’s behaviour but also as practical training sessions including debriefings, as simulation-based learning enhances the educational curriculum results such as clinical competency.^37^ A particular asset of our study regarding internal validity lies in its randomised, blinded, and pre-test-post-test design as well as in the inclusion of an NTS seminar group and a control group for analysing between-group effects.^38^ Unfortunately, this approach is often lacking in studies addressing seminar or training effects.^34^^,^^39^^,^^40^

### Future research

Our findings suggest that studies which include simulation sessions as practice sessions added to a single seminar design are desirable. This would enable an analysis of the number of practice sessions required to affect behaviour and patient treatment. It would also be interesting to analyse whether several simulation sessions including debriefings are superior to an NTS seminar on patient outcome. Such a study design might show that practical session and debriefed problem-based learning are required rather than theoretical inputs such as seminars. If this is the case, an adapted medical school curriculum might only contain well-designed and debriefed simulation sessions but no knowledge-based seminars.

The simulation sessions, and possibly the particular scenarios, functioned as stressors for the students. Since stress might enhance the retrieval of learned knowledge, but too much stress might decrease memory capacity,^41^^,^^42^ future studies should determine suitable simulation scenarios for the respective student cohorts so that the requirements do not overwhelm the student’s abilities. Furthermore, it might be desirable to measure cortisol concentrations as a more objective biological indicator of the stress responses.

Finally, it seems prudent to analyse effects of a more extensive NTS training on the performance of medical students when completing tasks of which they have better mastery. In this respect, a study on medical students in their internship year would appear to be most appropriate.

## Conclusions

In summary, although seminar-based NTS training demonstrated many positive sequelae, such training without practice appears to be only partially effective, as the NTS seminar showed no statistically significant improvement in simulated patient’s outcome. However, if such NTS courses are to be embedded into medical schools’ standard curricula to improve student’s handling of emergencies, a curricular design that combines practical simulation sessions with debriefings along with seminar sessions appears to be crucial.

### Conflicts of Interest

The authors declare that they have no conflict of interest.

## Supplementary material

Supplementary file 1 Appendix A. Overview of the non-technical skills observation system

Supplementary file 2 Appendix B. Overview of all collected medical data in Simulation I and Simulation II

Supplementary file 3 Appendix C. Demographic data and study variables at baseline measurement
