# Supplementary material for: Does teaching non-technical skills to medical students improve those skills and simulated patient outcome?
Source: Int J Med Educ. 2017 Mar 29;8:101–13. doi: 10.5116/ijme.58c1.9f0d (PMC5376493; doi:10.5116/ijme.58c1.9f0d)
Supplement: Supplementary file 1 — Appendix A. Overview of the non-technical skills observation system [file ijme-8-101-S1.pdf]

Appendix A

Overview of the non-technical skills observation system (behavioural markers are examples and therefore incomplete)

Category	Elements	Behavioural markers - General example items	Behavioural markers – Specific example items
**Situation awareness**	Gathering information	Procures information for anamnesis	Inquiry about previous illnesses, reason for hospital admission
	Recognising and understanding	Provides others with information about the patient's symptoms	"Patient has …!"
	Anticipating	Gives orders for possibly occurring problems	"If intubation impossible, mask ventilation."
**Task management**	Planning and preparing	Communicates plans to colleagues	"We will now …!" / "I will now…!"
	Prioritising	Discusses the important points with others	"First administration of oxygen, then stabilisation of the circulatory system."
	Providing and maintaining standards	Fulfils standards	Recognisable prioritisation/ positioning of devices
	Identifying and utilising resources	Recognises available resources and uses them	Delegation of actions (oxygen mask/ blood pressure measurement)
	Useful distribution of tasks	Can instruct employees	Corrects technique cardiac pressure massage, admission of oxygen, supervision
**Team working**	Co-ordinating activities with team members	Coordinates with others	No cardiac pressure massage with a simultaneous intubation
	Exchanging information	Speaks loudly and clearly	Expresses the instructions clearly and comprehensibly
	Using authority and assertiveness	Assumes responsibility and leadership	Keeps leading the team, remains as supervisor
	Assessing competencies	Checks the others' skills and knowledge earlier	"Can you do this measure? Have you done it before?"
	Supporting others	Informs the team after a difficult case and thanks them	Informs about the outcome of the intervention, "thank you for your support."
**Decision making**	Identifying options	Generates different options	"We could either do measure …, or measure..."
	Balancing risks and selecting options	Performs a selected option completely	Complete performance of the announced option
	Re-evaluating	Continues rethinking treatment options	"Another alternative would be …"
