# Supplementary material for: Does teaching non-technical skills to medical students improve those skills and simulated patient outcome?
Source: Int J Med Educ. 2017 Mar 29;8:101–13. doi: 10.5116/ijme.58c1.9f0d (PMC5376493; doi:10.5116/ijme.58c1.9f0d)
Supplement: Supplementary file 2 — Appendix B. Overview of all collected medical data in Simulation I and Simulation II [file ijme-8-101-S2.pdf]

Appendix B

Overview of all collected medical data in Simulation I and Simulation II

Simulation No.	Observed criteria
**Simulation I**	Understanding the gravity of the situation (yes/no)
	Taking the patient´s history quickly (yes/no)
	Checking airway, breathing and circulation (yes/no)
	Flattening the bed or using a reanimation board (yes/no)
	Continuing resuscitation measures (yes/no)
	Call for help (yes/no)
	Time until emergency call (seconds)
	Application of ERC guidelines (yes/no)
	Time until first rhythm analysis (seconds)
	No-flow-time from beginning of the scenario until continuing resuscitation measures (seconds)
	Providing pure oxygen ventilation (yes/no)
	Recognition of ventricular fibrillation (yes/no)
	Continuing chest compressions until administration of an electric shock (yes/no)
	Time until the first defibrillation (seconds)
	Administration of amiodarone after third unsuccessful defibrillation (yes/no)
	Time until first administration of amiodarone (seconds)
	Administration of epinephrine after third unsuccessful defibrillation (yes/no)
	Time until first administration of epinephrine (seconds)
	Repetition of epinephrine administration after 3 – 5 minutes (yes/no)
	Return of spontaneous circulation (yes/no)
	Time until return of spontaneous circulation (seconds)
	Using pulse oximetry (yes/no)
	Using (non-invasive) measurement of blood pressure (yes/no)
**Simulation II**	Progression of scenario to second phase "worsening anaphylaxis" (yes/no)
	Progression of scenario to last phase "severe anaphylaxis" (yes/no)
	Improving patient´s condition (yes/no)
	Time until improving patient´s condition (seconds)
	Taking the patient´s history quickly (yes/no)
	Diagnosing an "anaphylactic reaction" (yes/no)
	Time until diagnosing "anaphylactic reaction" (seconds)
	Making a wrong suspected diagnosis (yes/no)
	Call for help (yes/no)
	Time until emergency call (seconds)
	Stopping antibiotic infusion (yes/no)
	Time until stopping antibiotic infusion (seconds)
	Provided oxygen therapy via non-rebreathing mask (yes/no)
	Time until oxygen administration (seconds)
	Elevating position of the upper body (yes/no)
	Antihistamine administration (yes/no)
	Time until antihistamine administration (seconds)
	Initiation of blood volume expansion therapy (yes/no)
	Time until initiation of blood volume expansion therapy (seconds)
	Administration of epinephrine (yes/no)
	Time until first administration of epinephrine (seconds)
	Using pulse oximetry (yes/no)
	Time until connecting pulse oximetry (seconds)
	Using (non-invasive) measurement of blood pressure (yes/no)
	Time until first (non-invasive) measurement of blood pressure (seconds)
	Repetition of (non-invasive) measurement of blood pressure (yes/no)
	Securing respiratory track with endotracheal tube (yes/no)
