# Supplementary material for: Does teaching non-technical skills to medical students improve those skills and simulated patient outcome?
Source: Int J Med Educ. 2017 Mar 29;8:101–13. doi: 10.5116/ijme.58c1.9f0d (PMC5376493; doi:10.5116/ijme.58c1.9f0d)
Supplement: Supplementary file 3 — Appendix C. Demographic data and study variables at baseline measurement [file ijme-8-101-S3.pdf]

## Appendix C

Demographic data and study variables at baseline measurement (before seminar intervention)

| Variables | NTS Group Mean (SD) | Control Group Mean (SD) | Significance |
|---|---|---|---|
| Age | 25.98 (3.70) | 25.88 (3.26) | $t_{(75)}$ = 0.12, p = .91, r = 0.01 |
| Students` semester | 7.84 (1.05) | 8.21 (.48) | $t_{(75)}$ = -1.90, p = .06, r = 0.21 |
| **Presence** | | | |
| Presence | 3.55 (1.02) | 3.23 (1.00) | $t_{(75)}$ = 1.38, p = .17, r = 0.16 |
| **Stress** | | | |
| Stress | 2.75 (0.55) | 2.90 (0.39) | $t_{(74.25)}$ = -1.41, p = .16, r = 0.16 |
| **Attitude** | | | |
| Leadership | 3.23 (0.50) | 3.32 (0.34) | $t_{(75)}$ = -0.95, p = .35, r = 0.11 |
| Assertiveness | 2.81 (0.71) | 2.75 (0.68) | $t_{(75)}$ = 0.38, p = .70, r = 0.04 |
| Debriefing | 3.71 (0.40) | 3.56 (0.53) | $t_{(75)}$ = 1.42, p = .16, r = 0.16 |
| Feedback | 3.63 (0.54) | 3.59 (0.56) | $t_{(75)}$ = 0.32, p = .75, r = 0.04 |
| Realistic perception of stress | 2.76 (0.86) | 2.74 (0.59) | $t_{(75)}$ = 0.14, p = .89, r = 0.02 |
| Denial of stress | 1.92 (0.99) | 1.81 (.78) | $t_{(75)}$ = 0.53, p = .60, r = 0.06 |
| Dealing with mistakes | 2.49 (0.87) | 2.76 (0.63) | $t_{(75)}$ = -1.56, p = .12, r = 0.18 |
| Teamwork | 2.70 (0.63) | 2.71 (0.64) | $t_{(75)}$ = 0.06, p = .96, r = 0.01 |
| **NTS** | | | |
| Situation awareness | 1.17 (0.50) | 1.18 (0.43) | $t_{(75)}$ = -1.14, p = .89, r = 0.13 |
| Task management | 1.40 (0.65) | 1.30 (0.56) | $t_{(75)}$ = 0.69, p = .49, r = 0.08 |
| Teamwork | 1.37 (0.57) | 1.31 (0.53) | $t_{(75)}$ = 0.44, p = .66, r = 0.05 |
| Decision making | 0.90 (0.58) | 0.85 (0.58) | $t_{(75)}$ = 0.39, p = .70, r = 0.04 |

Range of scales: Presence from 0 to 5, Stress from 0 to 4, Attitudes from 0 to 4, and NTS from 0 to 3.
